# Focusing on Diabetic Ulcers

**Published:** 2020-02-20

**Authors:** C Setacci, D Benevento, G De Donato, E Viviani, UM Bracale, L del Guercio, G Palasciano, F Setacci

**Affiliations:** 1 Vascular Surgery Unit, University of Siena, Italy; 2 Vascular Surgery Unit, Department of Public Health, University Federico II of Naples, Naples, Italy; 3 Vascular Surgery Unit IRCCS Multimedia, Milano, Italy

**Keywords:** Diabetes mellitus, lower limb amputation, vascular surgery

## Abstract

Foot ulcers associated with Diabetes mellitus require immediate attention due to risk of amputation if left untreated. Herein we focus on the mitigating risk factors and physiopathology of the diabetic foot, recounting our own surgical approach and revascularization procedures.

## I. INTRODUCTION

Diabetes mellitus (DM) is one of the most common chronic diseases and largest global health emergencies of the 21st century. Prevalence of types 1 and 2 diabetes is on the rise and in 2015, 415 million adults worldwide were estimated to have had DM, including 193 million undiagnosed people and 318 million adults affected with impaired glucose tolerance; a condition that indicates high risk for developing the disease in the future. It is expected that if this number is not reduced, by 2040 up to 642 million people will be diagnosed with diabetes.^1^

Chronic diseases such as DM are causing increasing numbers of deaths worldwide. In 2015 of 56,4 million deaths across the globe diabetes, which represents the sixth leading cause of global deaths in 2015, killed 1.6 million people, up from fewer than 1 million in 2000.^2^ Microvascular (neuropathy, retinopathy, nephropathy) and macrovascular (cerebral, myocardic, peripheral), all chronic complications of diabetes, are a major cause of premature death, reduced quality of life and disability.

The World Health Organization defines diabetic foot as an infection, ulceration or the destruction of deep tissues of the foot associated with nephropathy and/or peripheral arterial disease.^3^

The 2008 Consensus document of the international working group on the diabetic foot reports the following statements.^4^

- Foot problems are common, very expensive, and life threatening.
- Every 30 seconds a lower limb is lost somewhere in the world as a consequence of diabetes.
- Up to 70% of all leg amputations are performed on people with diabetes.
- Up to 85% of all amputations are preceded by an ulcer.
- In developed countries, up to 5% of people with diabetes have a diabetic foot ulcer
- 12% to 15% of health care resources are used for diabetes.

More attention is being paid to the quality of life of patients with diabetic foot ulcers and amputees whose quality of life is diminished at the same rate as that for patients with cancer or those with chronic kidney disease undergoing dialysis 17. No doubt the lives of people after amputation are profoundly affected. Many are unable to work, become dependent on others and cannot pursue an active social life. In developing countries, the situation is even worse because a whole family may have no income if the active member is suffering from a chronic ulcer or has undergone amputation.

## II. PHYSIOPATHOLOGY

The Physiopathology of the diabetic foot is rather complex. If diabetes is not controlled for several years, complications frequently arise. Vasculopathy and neuropathy are the most important complications leading to diabetic foot problems. Small traumas can have devastating effects. As primary care physicians treating diabetes, we need to consider three important factors: vasculopathy/neuropathy/trauma will lead to an ulcer. Infection increases the chances of amputation, especially if the patient has poor peripheral circulation.

Diabetic peripheral neuropathy is characterized by the involvement of all fibers (sensory, motor and autonomic). Motor neuropathy results in atrophy and weakness of the muscles of the foot, leg and joint rigidity with consequent abnormal walking patterns resulting in an increase of peek pressure on the plantar aspect of the foot.

As a consequence of muscle impairment and joint rigidity foot deformities may cause ulcers on both the plantar and dorsal sides of the foot. Autonomic neuropathy results in reduced or absent sweating leading to dry skin with cracks and fissures.

## III. RISK FACTOR MODIFICATION

Cardiovascular morbidity and mortality are markedly increased in patients with peripheral artery disease. Therefore, treatment of neuroischemic ulcers should not be solely focused on the foot but should also aim to reduce poor survival rates. In patients without diabetes, cessation of smoking has been shown to decrease the risk of developing intermittent claudication and decrease the subsequent risk of amputation. Moreover, if the patient stops smoking, patency rates for vascular reconstruction are higher and the risk of death is lower.

Although there are no studies that can demonstrate that treating hypertension and dyslipidemia have any beneficial effects.

The involvement of sympathetic nerve fibers causes increased arteriovenous shunting which leads to a overheated and edematous foot with distended dorsal foot veins.

Sensory neuropathy may reduce pain perception, increasing the risk of ulceration secondary to repetitive trauma during walking^8^–^10^. Insensitive callus on the plantar foot surface caused by peak of pressure frequently anticipates the onset of a neuropathic plantar ulcers 5,6,11.

Charcot neuro-osteoarthropathy is a devastating complication of diabetic foot syndrome. Acute Charcot usually presents with a hot, inflamed, swollen and sometimes painful foot without skin lesions - clear differentiation from infection is mandatory to ensure appropriate management. Progression is often rapid with bone fragmentation and joint destruction followed by a longitudinal arch of the foot is a common evolution leading to typical “rocker bottom” deformity with a high risk of deep infection ulcers.^11^

## IV. SURGICAL TREATMENT

### Surgical treatment of infected superficial ulcers of the foot

Debridement of an infection of the superficial ulcer consists of removal of a necrotic or non-vital tissue with exposure of a healthy and bleeding wound bed. Once the infected and/or necrotic tissue has been removed, negative pressure wound therapy is generally used to guarantee the cleansing of the wound bed and the quick removal of all exudates which could still be contaminated by residual deep bacterial colonies.

### Surgical treatment of deep infection of the foot

The infectious process of a diabetic foot can start directly with the involvement of the deep structure of the foot but very often represents a late complication of a superficial ulcer or wet gangrene not correctly treated. From a clinical point of view, infection of the foot can be classified as follows:

1. Phlegmon
2. Infective compartment syndrome
3. Necrotizing fasciitis

#### Gangrene –

Gangrene represents the final evolution in insufficient blood perfusion of the tissue. While dry gangrene doesn’t represent an emergent clinical status due lack of infection, wet gangrene, which is often the final evolution of an infected superficial ulcer in the presence of CLI, needs to be treated immediately to avoid further worsening of the infection.

## V. REVASCULARIZATION PROCEDURE

Revascularization is a key therapy in ischemic diabetic lesions because re-establishing an adequate blood supply to the wound is essential for avoiding major amputation^12^.

1. Our first approach is an “angioplasty first” strategy however in the face of extremely long femoro-popliteal and/or below the knee vessel obstruction, the correct decision is not simple.^13^ In these cases the patient’s clinical status, foot lesion, suitability of veins and of a correct landing zone for distal bypass must be considered. Percutaneous revascularization doesn’t need general anesthesia and can be easily carried out in high surgical risk patients due to age/comorbidities and reduced life expectancy. Percutaneous revascularization can be divided into multiple steps and be repeated in case of restenosis. It can be performed in foot lesions involving distal anastomosis sites, in case of absence of adequate veins or if distal bypass surgery guarantees superior long-term patency.
2. Targets of revascularization therapy: complete revascularization is better than partial revascularization.^14^–^15^ The first principle guiding our revascularization strategy must be to give to the foot the best possible blood supply. Another emerging concept is the “wound related artery” revascularization.^16^ Following the angiosome concept, a successful angioplasty or bypass of the artery directly feeding the wound region leads to higher rates of limb salvage and wound healing.
3. We try to perform the angiographic study and the percutaneous revascularization in the same session. The procedure must be tailored to technically realistic strategies and on general patient status.
